# D-Stellate Neurons of the Ventral Cochlear Nucleus Decrease in Auditory Nerve-Evoked Activity during Age-Related Hearing Loss

**DOI:** 10.3390/brainsci9110302

**Published:** 2019-10-31

**Authors:** Yong Wang, Meijian Wang, Ruili Xie

**Affiliations:** 1 Department of Otolaryngology, The Ohio State University, Columbus, OH 43210, USA; yong.wang@osumc.edu (Y.W.); meijian.wang@osumc.edu (M.W.); 2 Department of Neuroscience, The Ohio State University, Columbus, OH 43210, USA

**Keywords:** age-related hearing loss, D-stellate, auditory nerve, firing rate, synaptic transmission, EPSC, intrinsic property

## Abstract

Age-related hearing loss (ARHL) is associated with weakened inhibition in the central auditory nervous system including the cochlear nucleus. One of the main inhibitory neurons of the cochlear nucleus is the D-stellate neuron, which provides extensive glycinergic inhibition within the local neural network. It remains unclear how physiological activities of D-stellate neurons change during ARHL and what are the underlying mechanisms. Using in vitro whole-cell patch clamp technique, we studied the intrinsic membrane properties of D-stellate neurons, the changes of their firing properties, and the underlying mechanisms in CBA/CaJ mice at the ages of 3–4 months (young), 17–19 months (middle age), and 27–33 months (aged). We found that the intrinsic membrane properties of D-stellate neurons were unchanged among these three age groups. However, these neurons showed decreased firing rate with age in response to sustained auditory nerve stimulation. Further investigation showed that auditory nerve-evoked excitatory postsynaptic currents (EPSCs) were significantly reduced in strength with age. These findings suggest that D-stellate neurons receive weakened synaptic inputs from the auditory nerve and decreased sound driven activity with age, which are expected to reduce the overall inhibition and enhance the central gain in the cochlear nucleus during ARHL.

## 1. Introduction

The cochlear nucleus (CN) is the first neural station in the central auditory pathway that processes all sound information from the periphery auditory nerve and projects to higher auditory nuclei [1]. It is widely observed that auditory processing in CN neurons is shaped by extensive inhibition [2,3,4,5,6,7], which is predominantly mediated by glycine [8,9]. During aging, inhibition diminishes in the CN, with changes that include decreased glycine levels [10], modified glycine receptors [11], reduced inhibitory synaptic drive [12], and weakened inhibitory function [13]. However, it remains unclear how physiological properties of CN inhibitory neurons change during aging, as well as the underlying mechanisms. 

One of the major CN inhibitory interneurons is the D-stellate neuron in the ventral cochlear nucleus (VCN) [14,15,16,17,18]. They receive excitatory inputs from the auditory nerve, and provide glycinergic inhibition locally within the VCN and to the dorsal cochlear nucleus (DCN) [5,17,19,20,21], as well as to the contralateral CN [15,22,23]. In response to tones, D-stellate neurons fire sustained action potentials throughout the duration of the stimulus with regular inter spike interval only at the stimulus onset, which manifest as an onset chopper peristimulus time histogram (PSTH) [19,24,25,26,27]. Consistent with their widespread dendritic arbors that across multiple iso-frequency bands [18,19,26,28,29], D-stellate neurons are broadly tuned and respond well to broadband stimuli like noise [25,27]. These response features enable D-stellate neurons to provide broad band inhibition to shape the sound processing of their target neurons [30], including bushy and T-stellate neurons of the VCN [5] where glycinergic inhibition helps improve the temporal precision of encoded information [2,31]. Our previous study showed that during age-related hearing loss (ARHL), glycinergic inhibition from the DCN to bushy neurons was weakened because of increased failure rate and decreased quantal content at the synapse [12]. However, little is known about how inhibitory interneurons of the CN, particularly the D-stellate neurons, change with age that contribute to weakened inhibition and ultimately ARHL. 

In this study, we investigated the physiological changes of the D-stellate neurons in VCN during ARHL in three age groups of CBA/CaJ mice at 3–4 months (young with normal hearing), 17–19 months (middle age with mild hearing loss), and 27–33 months (aged with ARHL). We found that the D-stellate neurons had similar intrinsic membrane properties among all three age groups of mice, suggesting that these inhibitory interneurons are intrinsically normal in function during aging. However, D-stellate neurons showed decreased firing rate with age in response to long trains of auditory nerve stimulation. Voltage clamp experiments further showed that the synaptic drive from the auditory nerve to D-stellate neurons was significantly weakened with age. The findings suggest that sound driven activity in D-stellate neurons is decreased during aging, which contributes to the weakened inhibition in the CN. The decreased firing rate in D-stellate neurons is the result of weakened synaptic drive from the auditory nerve during ARHL. 

## 2. Materials and Methods

All experiments were performed under the guidelines of the protocols approved by the Institutional Animal Care and Use Committee at The Ohio State University.

### 2.1. Animals

CBA/CaJ mice of either sex were used at three age groups: 3–4 months (young; *n* = 19), 17–19 months (middle age; *n* = 20), and 27–33 months (aged; *n* = 25). Mice were initially purchased from the Jackson Laboratory, bred and maintained at the university animal facility until the age of experimental use.

### 2.2. Auditory Brainstem Response (ABR)

The hearing status of the mice was evaluated by recording ABRs as previously described [32,33]. Mice were anesthetized with intraperitoneal injection of ketamine (100 mg/kg) and xylazine (10 mg/kg), and were placed on a heating pad to maintain the body temperature inside a custom-made sound-attenuating box. ABR recordings were performed using a RZ6-A-P1 bioacoustic system with BioSigRZ software (Tucker-Davis Technologies). Clicks (0.1 ms, monophasic with alternating phase) were played at sound levels from 20 to 90 dB SPL, through a free-field MF1 speaker (Tucker-Davis Technologies) positioned 10 cm away from the measured ear. Three needle electrodes were placed at the ipsilateral pinna, vertex, and the rump, respectively, to collect the click-evoked ABR signals with 512 repetitions per sound level. Hearing threshold was defined as the minimum sound level that elicited recognizable ABR waves, and was determined in all mice with observers blinded to animal age during the analysis.

### 2.3. Acute Brain Slice Preparation

After ABR recordings, mice were decapitated and brainstems dissected out. Parasagittal slices containing cochlear nucleus were cut at the thickness of 350 µm using a Vibratome 1000 (Technical Products, Inc., St Louis, MO, USA) or a VT1200S Microtome (Leica Biosystems, Wetzlar, Germany) [32,33]. The brain slices were then incubated in artificial cerebral spinal fluid (ACSF) for 45 min before electrophysiological recording began. ACSF contained (in mM): 122 NaCl, 3 KCl, 1.25 NaH_2_PO_4_, 25 NaHCO_3_, 20 glucose, 3 *myo*-inosital, 2 sodium pyruvate, 0.4 ascorbic acid, 1.8 CaCl_2_, and 1.5 MgSO_4_, was gassed with 95% O_2_ and 5% CO_2_ and pre-warmed to 34 °C. The same ACSF solution was used for slicing, incubating, as well as electrophysiological recording throughout the experimental process. 

### 2.4. Electrophysiological Recording

Cochlear nucleus slice was transferred to the recording chamber under Axio Examiner microscope (Carl Zeiss, Oberkochen, Germany), submerged in ACSF bath with a constant flow of 2–3 mL/min and the temperature controlled at 34 °C throughout the recording session. Recording pipettes were pulled from borosilicate glass (KG-33; King Precision Glass, Claremont, CA, USA) with a Sutter P2000 puller (Sutter Instrument, Novato, CA, USA). Data acquisition was made using a Multiclamp 700 B amplifier, Digidata 1550 B acquisition system, and pClamp 11 software (Molecular Devices). Whole-cell patch clamp recording was performed under either current clamp or voltage clamp mode. For current clamp recordings, glass pipettes were filled with K-gluconate-based electrode solution that contained (in mM): 126 K gluconate, 6 KCl, 2 NaCl, 10 HEPES, 0.2 EGTA, 4 MgATP, 0.3 GTP, and 10 Tris-phosphocreatine, with pH adjusted to 7.20. For voltage clamp recordings, glass pipettes were filled with Cs-MetSO_3_-based electrode solution that contained (in mM): 130 CsMetSO_3_, 5 CsCl, 5 EGTA, 10 HEPES, 4MgATP, 0.3 GTP, 10 Tris-phosphocreatine, and 3 QX-314. To activate the auditory nerve inputs, electric pulse stimulation was delivered through a 75-µm diameter concentric stimulating electrode (Fredrick Haer Company, Bowdoin, ME, USA), which was placed at the area of the auditory nerve root. Two µM strychnine was added to the ACSF bath to block glycinergic inhibitory transmission in all recordings. Membrane potentials from current clamp recordings were adjusted with a junction potential of −12 mV. 

### 2.5. Cell Identification

All electrophysiological recordings were made from the anteroventral cochlear nucleus (AVCN), where three types of neurons were encountered in our experiments including bushy, T-stellate, and D-stellate neurons. Only D-stellate neurons were included in this study based on their distinctive features in morphology and electrophysiology [29]. Alexa Fluor 488 dye was included in the electrode solution to fill every recorded neuron in order to visualize cellular morphology during or immediately after the recording. D-stellate neurons were identified based on: (1) Having long and extensive dendrites that expand across multiple auditory nerve fascicles [28,29]; (2) fired tonic spikes throughout the duration of the supra-threshold current step injections; and (3) showed fast depolarization sag to hyperpolarizing current injections [29,34,35]. Additionally, D-stellate neurons under voltage clamp recordings responded with slow eEPSCs to auditory nerve inputs [29]. 

### 2.6. Data Analysis

Data were analyzed using Igor Pro software (version 6.37; Wave Metrics, Lake Oswego, OR, USA) with custom-written functions. Intrinsic membrane properties were analyzed from all D-stellate neurons with current clamp data, as described in previous studies [29,32]. The resting membrane potential was calculated as the average membrane potential during a 10-ms window preceding current step injections (Figure 2A–C). Membrane input resistance was calculated as the slope of the current-voltage relationship curve from four smallest hyperpolarizing current step injections. Membrane time constant was determined as the average time constant from single exponential fits of the same four hyperpolarizing responses from the onset to the negative peak. Threshold current was defined as the minimum level of depolarizing current needed to trigger the first action potential in the recorded neuron. To quantify the strength of hyperpolarization activated depolarization sag, a sag ratio (b/a) was calculated, in which a and b were the peak amplitude and the steady-state amplitude of the hyperpolarization, respectively [29,34]. The decay time constant of the depolarization sag was derived from the single exponential fit of the hyperpolarizing trace from the peak to the end of the current step injection [29]. Spike amplitude was the voltage difference between the resting membrane potential and the spike peak. Spike half-width was the duration of the spike at 50% of spike amplitude. Spike threshold was defined as the membrane voltage where the peak of the second derivative was found during the rising phase of the action potential [29]. Vector strength was calculated from the spike trains to evaluate the temporal precision of spike timing as previously described [29,32,36].

GraphPad Prism (version 6.0h; GraphPad Software, San Diego, CA, USA) was used in all statistical analyses. Data were presented as mean ± standard deviation.

## 3. Results

### 3.1. Auditory Brainstem Responses (ABRs) Reveal Age-Related Hearing Loss

**Figure 1:**
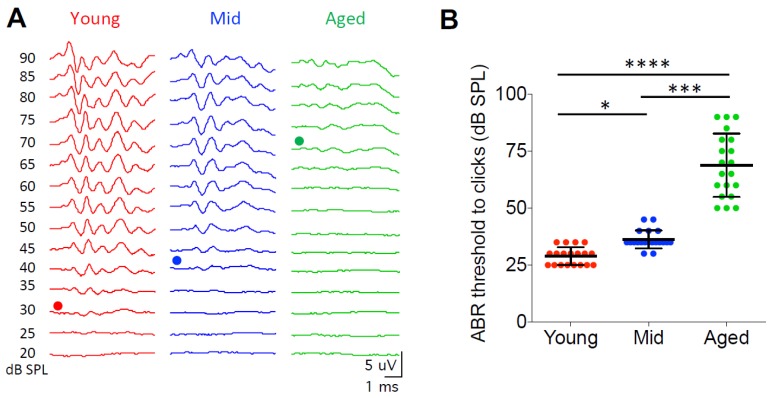
Auditory brainstem responses reveal age-related hearing loss in CBA/CaJ mice. (**A**) Representative auditory brainstem response (ABR) waveforms from mice in three age groups in response to clicks. Dots mark the traces at hearing threshold. (**B**) Summary ABR thresholds from all mice. Kruskal–Wallis test: * *p* < 0.05; *** *p* < 0.001; **** *p* < 0.0001. Individual data points are shown along with mean ± S.D. for each group.

In order to test the hearing status of the CBA/CaJ mice in this study, we measured the ABRs from all mice in three age groups to clicks at sound levels from 20 to 90 dB SPL. Hearing threshold was determined as the minimum sound level that evoked identifiable ABR waveforms as shown in Figure 1A. Young mice showed an average hearing threshold of 29.0 ± 3.9 dB SPL (*n* = 19), which was significantly lower than the thresholds of the middle age (36.3 ± 3.9 dB SPL; *n* = 20) and the aged mice (68.8 ± 13.9 dB SPL; *n* = 20) (Figure 1B; Kruskal–Wallis test: *p* < 0.0001; Dunn’s multiple comparisons test: *p* < 0.05 between young and middle age, *p* < 0.0001 between young and aged, *p* < 0.001 between middle age and aged). Hearing thresholds could not be determined from five aged mice because no clear ABR waveforms were evoked by clicks at even the highest tested level of 90 dB SPL. These five mice were not included in Figure 1 and the ABR analysis. The observed changes in hearing threshold with age in this study are consistent with previous reports about this mouse strain [37,38].

### 3.2. D-Stellate Neurons Show No Change in Intrinsic Membrane Properties with Age

**Figure 2:**
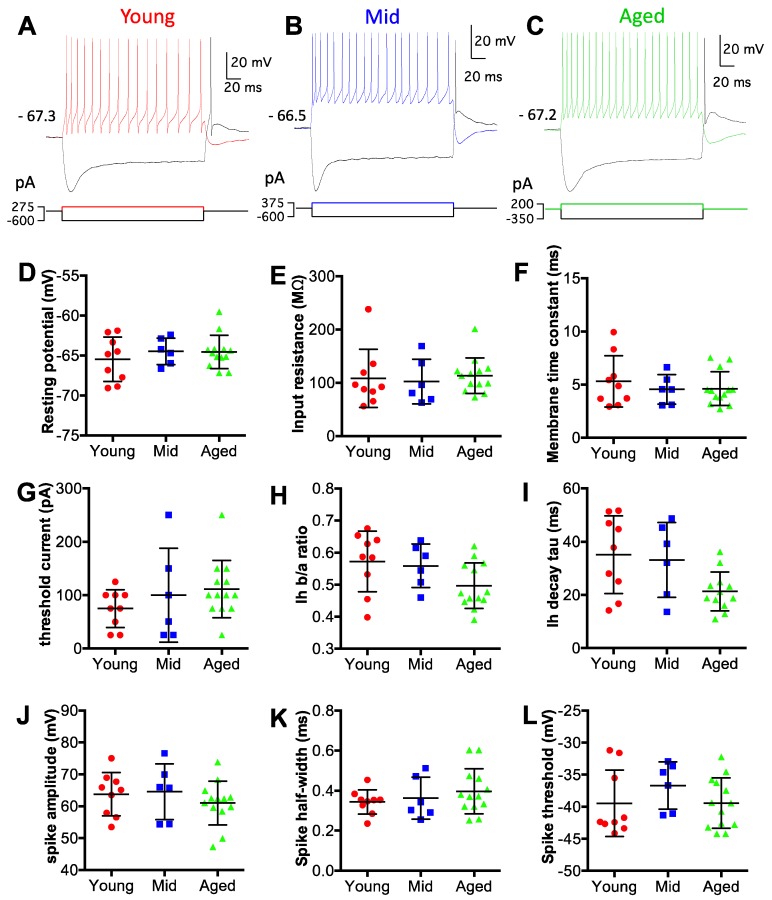
D-stellate neurons show no change in intrinsic membrane properties with age. (**A**–**C**) Example membrane responses of D-stellate neurons to current injections from three age groups. Numbers mark the resting membrane potentials in mV. Bottom panels: current pulse injections. (**D**–**L**) Summary plots of all D-stellate neurons in resting membrane potential (**D**), membrane input resistance (**E**), membrane time constant (**F**), threshold current injection to trigger spikes (**G**), depolarization sag steady state versus peak ratio (**H**), depolarization sag decay time constant (**I**), spike amplitude (**J**), spike halfwidth (**K**) and spike threshold voltage (**L**). Kruskal–Wallis test (non-parametric one-way ANOVA) showed no significant difference in all measurements in (**D**–**L**) (*p* > 0.05).

To evaluate the intrinsic membrane properties of D-stellate neurons, we performed whole-cell patch clamp recording under current clamp mode using acute brain slices from mice at all three age groups. D-stellate neurons were identified based on their morphological and electrophysiological features as described in Materials and Methods. Data were collected from 9 D-stellate neurons from the young mice, 6 from the middle age mice, and 13 from the aged mice. Intrinsic membrane properties of all neurons were measured from current step injection responses (Figure 2A–C; see Materials and Methods for details). As shown in Figure 2D–L, no significant difference was found in any of the nine different properties among the three age groups. Specifically, the average resting membrane potentials of D-stellate neurons were −65.5 ± 2.8 mV (young), −64.5 ± 1.7 mV (middle age), and −64.6 ± 2.1 mV (aged) (Figure 2D; Kruskal–Wallis test: *p* = 0.690). The average membrane input resistances were 108 ± 54 MΩ (young), 102 ± 42 MΩ (middle age), and 113 ± 33 MΩ (aged) (Figure 2E; Kruskal–Wallis test: *p* = 0.473). The membrane time constants were 5.3 ± 2.4 ms (young), 4.6 ± 1.4 ms (middle age), and 4.6 ± 1.6 ms (aged) (Figure 2F; Kruskall–Wallis test: *p* = 0.857). The threshold currents, which were the minimum current injection level that evoked action potentials, were 75 ± 35 pA (young), 100 ± 88 pA (middle age), and 112 ± 54 pA (aged) (Figure 2G; Kruskall–Wallis test: *p* = 0.289). The magnitude of depolarization sag in response to hyperpolarizing current injection was quantified as the ratio of hyperpolarization levels at the steady state versus the peak (Ih b/a ratio). In three groups mice, the ratios were 0.57 ± 0.09 (young), 0.56 ± 0.07 (middle age), and 0.50 ± 0.07 (aged), respectively (Figure 2H; Kruskall–wallis test: *p* = 0.088). The decay time constants of the depolarization sags were 35 ± 15 ms (young), 33 ± 14 ms (middle age), and 21 ± 7 ms (aged) (Figure 2I; Kruskal–Wallis test: *p* = 0.055). The average spike amplitudes were 64 ± 7 mV (young), 65 ± 9 mV (middle age), and 61 ± 7 mV (aged) (Figure 2J; Kruskal–Wallis test: *p* = 0.433). The average spike half-widths were 0.34 ± 0.06 ms (young), 0.36 ± 0.11 ms (middle age), and 0.40 ± 0.11 ms (aged) (Figure 2K; Kruskal–Wallis test: *p* = 0.556). The average spike thresholds were −39.4 ± 5.2 mV (young), −36.7 ± 3.7 mV (middle age), and −39.4 ± 3.9 mV (aged) (Figure 2L; Kruskal–Wallis test: *p* = 0.319). In summary, D-stellate neurons from three age groups of mice showed no change in their intrinsic membrane properties, which suggests that these neurons themselves are functionally stable during aging. 

### 3.3. D-Stellate Neurons Decrease in Firing Rate to Auditory Nerve Inputs during Aging

**Figure 3:**
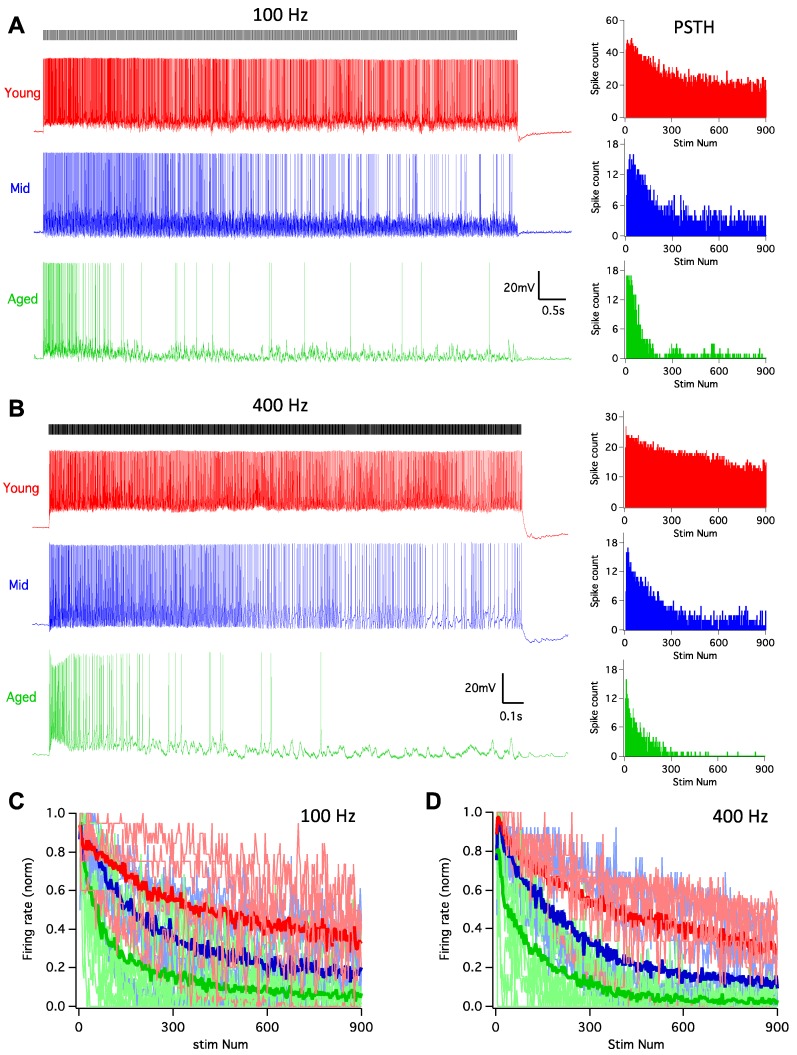
D-stellate neurons decrease in firing rate to auditory nerve inputs during aging. (**A**) Left: example responses of D-stellate neurons to auditory nerve inputs at 100 Hz. Ticks on top mark the timing of 900 stimuli at the auditory nerve. Note the decreased firing of spikes in middle and aged cells. Right: PSTH plots of the responses from the cells in left panels. (**B**) Same as in (**A**) except that the stimulus trains were at 400 Hz. (**C**) Normalized firing rate to 100 Hz stimulus trains in all D-stellate neurons from three age groups. Thin lines: individual neurons; thick lines: average firing rate in three age groups. (**D**) Normalized firing rates to 400 Hz stimulus trains.

**Figure 4:**
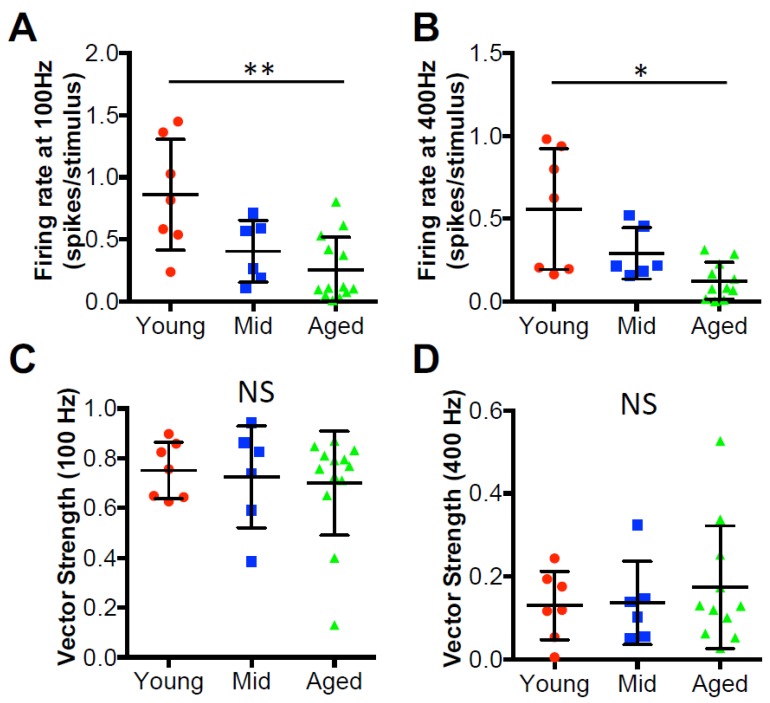
D-stellate neurons show decreased firing rate with age but no change in temporal coding. (**A**) Firing rates in all D-stellate neurons to 100 Hz stimulus trains at the auditory nerve. (**B**) Firing rates in all D-stellate neurons to 400 Hz stimulus trains at the auditory nerve. (**C**) Calculated vector strength of D-stellate spikes evoked by 100 Hz stimulus trains at the auditory nerve. (**D**) Calculated vector strength of D-stellate spikes evoked by 400 Hz stimulus trains at the auditory nerve. Kruskal–Wallis test (non-parametric one-way ANOVA): ** *p* < 0.01; * *p* < 0.05; NS: *p* > 0.05.

It is well-known that the auditory system is highly active, with auditory nerve fibers that can sustain firing at rates up to hundreds of Hz [39,40]. As one of the targets of the auditory nerve, D-stellate neurons are driven by such high rate innervation before providing inhibition to subsequent neurons to shape the processing of sound. In order to assess the functional changes of D-stellate neurons during aging, we examined their firing responses to auditory nerve inputs, which were activated by 900-pulse train stimulation at 100 and 400 Hz. In young mice, pulse train stimulation at 100 Hz evoked tonic spikes that lasted throughout the duration of the train (Figure 3A), with the PSTH plot revealing that the firing rate slightly decreased beyond the beginning of the train. In middle age mice, the response pattern was similar to that in young mice, except that the spike rate decreased more after the beginning of the train. The decrease in spike rate was even more profound in aged mice, and the firing pattern to the stimulus train became transient with few evoked spikes during the latter session of the trains. The firing rates of all D-stellate neurons in three age groups are plotted in Figure 3C, normalized to the maximum firing rates of the 100 Hz trains. Two-way ANOVA revealed significant decrease in firing rate with increased stimulus number (*p* < 0.0001) and age (*p* < 0.0001), with no interaction effect between the two (*p* > 0.9999). Similar changes in firing patterns with age were also observed in D-stellate responses to 400 Hz stimulus trains (Figure 3B) and summarized in Figure 3D (Two-way ANOVA: stimulus number effect: *p* < 0.0001; age effect: *p* < 0.0001; interaction: *p* > 0.9999). The actual firing rates to 100 Hz stimulus trains were 0.86 ± 0.45 spikes/stimulus in young mice, 0.40 ± 0.25 spikes/stimulus in middle age mice, and 0.26 ± 0.26 spikes/stimulus in aged mice (Figure 4A; Kruskal–Wallis test: *p* = 0.008); and the firing rates to 400 Hz stimulus trains were 0.56 ± 0.36 (young), 0.29 ± 0.16 (middle age), and 0.13 ± 0.11 spikes/stimulus (aged), respectively (Figure 4B; Kruskal–Wallis test: *p* = 0.014).

Our previous study showed that the spike timing of D-stellate neurons encodes temporal information of auditory nerve inputs at low rates (50–100 Hz) but not at high rates (200–400Hz) [29]. To test if there is any age-related deterioration in the encoding of temporal information in D-stellate neurons, we calculated the vector strength (see Materials and Methods) of the spike trains evoked by auditory nerve stimulation at 100 and 400 Hz. At 100 Hz, the average vector strength was 0.75 ± 0.11 (young), 0.72 ± 0.20 (middle age), and 0.70 ± 0.21 (aged), with no significant difference among three age groups (Figure 4C; Kruskal–Wallis test: *p* = 0.964). The spike temporal precision was also similar in D-stellate neurons at 400 Hz among different age groups, with the vector strength of 0.13 ± 0.08 (young), 0.14 ± 0.10 (middle age), and 0.17 ± 0.14 (aged), respectively (Figure 4D; Kruskal–Wallis test: 0.909). The results suggest that despite the decreased spike rate with increased age, there is no change in the temporal encoding of auditory nerve information in D-stellate neurons during aging. 

The findings that D-stellate neurons do not change in their intrinsic membrane properties during aging, but decrease in firing rate to long trains of auditory nerve inputs suggest that the excitatory synaptic drive is weakened with age in D-stellate neurons.

### 3.4. Synaptic Inputs to D-Stellate Neurons under Quiescence Show No Change during Aging

**Figure 5:**
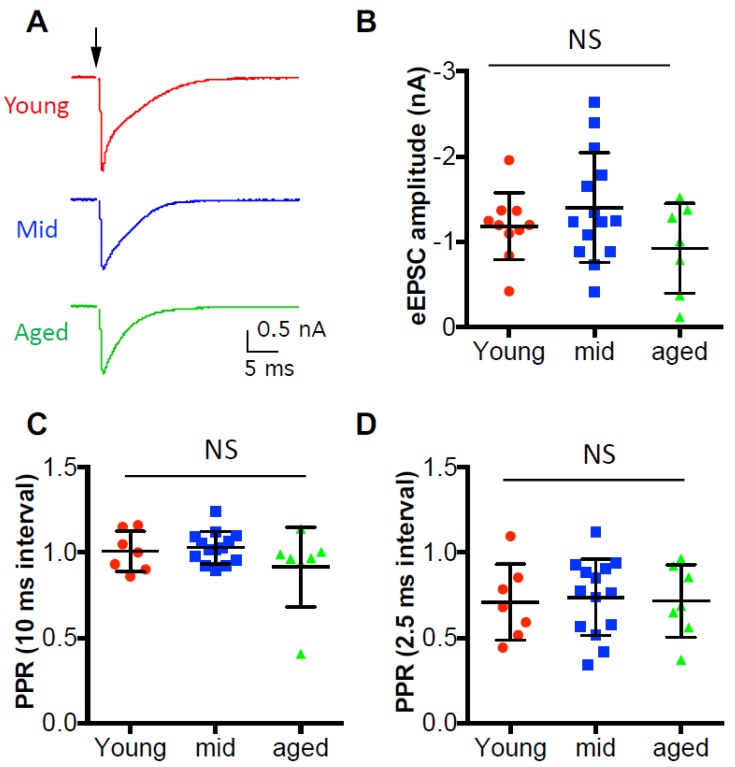
Synaptic inputs to D-stellate neurons under quiescence show no change during aging. (**A**) Single stimulation at the auditory nerve evoked excitatory postsynaptic current (EPSCs) in three example D-stellate neurons from three age groups. Arrow: stimulus onset. (**B**) Summary plot of the EPSC amplitude in all three groups. (**C**) Paired pulse ratio (PPR) of EPSC amplitudes at 10 ms interval. (**D**): Paired pulse ratio of EPSC amplitude at 2.5 ms interval. NS: not significant (Kruskal–Wallis test: *p* > 0.05).

In order to evaluate the synaptic strength of the auditory nerve inputs, we first recorded the evoked excitatory postsynaptic current (eEPSC) from D-stellate neurons under voltage clamp mode in response to single auditory nerve stimulation (Figure 5A). The average eEPSC amplitudes were measured from all D-stellate neurons and were 1.18 ± 0.39 nA in young mice, 1.40 ± 0.64 nA in middle age mice, and 0.92 ± 0.53 nA in aged mice (Figure 5B). No significant difference was found in eEPSC amplitude among three age groups (one-way ANOVA: *p* = 0.182), which suggests that the synaptic strength of the auditory nerve terminals onto D-stellate neurons do not change with age under quiescence. We also recorded the paired pulse stimulation evoked eEPSCs, and found no significant difference in paired pulse ratio at either 10-ms pulse interval (Figure 5C; Kruskal–Wallis test: *p* = 0.549) or 2.5-ms pulse interval (Figure 5D; Kruskal–Wallis test: 0.918). The results indicate that the initial release probability remains unchanged with age at the auditory nerve synapses onto D-stellate neurons. 

### 3.5. Synaptic Inputs to D-Stellate Neurons are Weakened with Age during Sustained Activities

**Figure 6:**
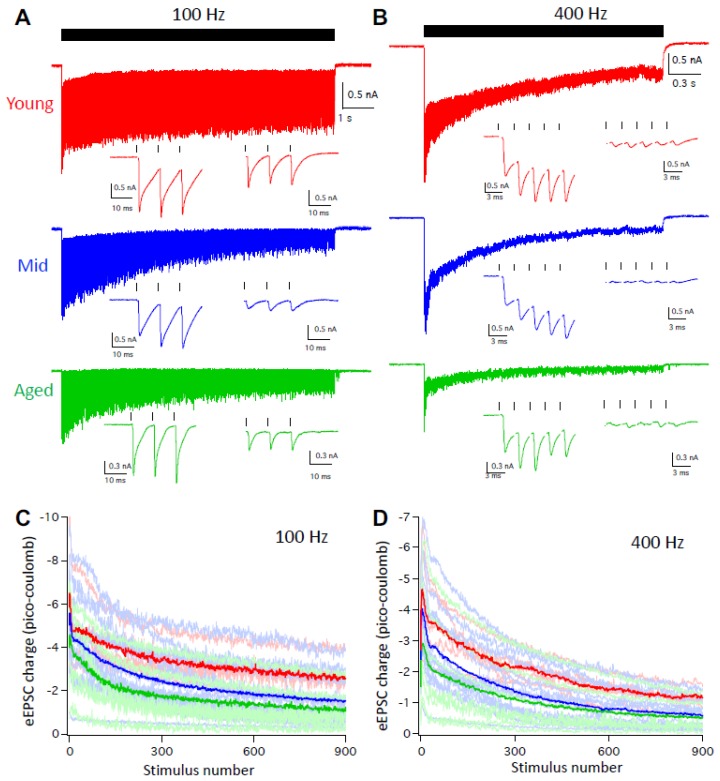
D-stellate neurons receive weakened synaptic inputs at high rate auditory nerve stimulation during aging. (**A**) Auditory nerve evoked EPSC trains at 100 Hz in D-stellate neurons from three age groups. Ticks on top mark the timing of 900 stimulus train. Two insets show evoked EPSCs by the first and last three stimuli of the train, respectively. (**B**) Auditory nerve-evoked EPSC trains at 400 Hz in the same D-stellate neurons as in (**A**). Traces in (**A**) and (**B**) are averages of 5 trials. (**C** and **D**) summary plots of EPSC charge transfer in D-stellate neurons at 100 (**C**) and 400 Hz (**D**). Thin lines are individual neurons; thick lines are the averages of all neurons in three age groups (red: young; blue: middle age; green: aged).

Given that the auditory system is highly active under physiological condition and D-stellate neurons decrease in firing with age at high rate activities (Figure 3 and Figure 4), we further evaluated the function of the auditory nerve synapses onto D-stellate neurons by recording the eEPSCs in response to trains of auditory nerve stimulation at 100 and 400 Hz. As shown in Figure 6A, 900-pulse stimulation at 100 Hz evoked reliable EPSCs in D-stellate neurons from young mice throughout the stimulus train, with reduced eEPSC amplitude beyond the onset because of synaptic depression. In both middle age and aged mice, D-stellate neurons responded with similarly reliable eEPSCs except that eEPSCs showed more synaptic depression throughout the duration of the stimulus train (Figure 6A). At 400 Hz, eEPSCs in D-stellate neurons from young mice showed more synaptic depression than 100Hz (Figure 6A,B, red traces). In contrast, both middle age and aged mice showed profound synaptic depression throughout the stimulus train with barely identifiable individual eEPSCs toward the latter session of the trains (Figure 6B, blue and green trace). Consistent with our previous study [29], eEPSCs in D-stellate neurons are slow in kinetics and show prominent temporal summation throughout the trains, which help drive the repetitive firing of spikes in these neurons. The summation of eEPSCs were greatly reduced in middle age and aged mice (Figure 6A,B), especially at 400 Hz (Figure 6B). To quantify the synaptic drive during the stimulus trains, we calculated the EPSC charge transfer throughout the 900-pulse trains. On average, eEPSC charges were the highest in young mice and the lowest in aged mice throughout the 100 Hz stimulus train (Figure 6C). Two-way ANOVA showed that the eEPSC charges were significantly decreased with age (*p* < 0.0001) and with stimulus number (*p* < 0.0001), with no interaction between the two (*p* > 0.9999). Similar differences were found in eEPSC trains at 400 Hz (Figure 6D; two-way ANOVA: *p* < 0.0001 for both age effect and stimulus number effect; interaction: *p* > 0.9999). 

The results showed that during aging, D-stellate neurons receive weakened sensory inputs from the auditory nerve with reduced synaptic drive, and fire less spikes during sustained activities. The decreased firing in D-stellate neurons would reduce the overall inhibition within the neural network and promote the response of other CN neurons to auditory nerve inputs. 

## 4. Discussion

One of the prevailing theories in brain aging is that inhibition diminishes with age, which leads to compromised neural function, including in the auditory system [41]. In CN, despite various reports about age-related changes of glycinergic inhibition in multiple disciplines [10,11,12,13], it remains unclear whether and how CN inhibitory neurons change physiologically during ARHL. In this study, we investigated the inhibitory D-stellate neurons of the VCN and found that these neurons do not change their intrinsic membrane properties with age, and thus are functionally stable during ARHL. However, aged D-stellate neurons show significantly reduced auditory nerve evoked activity, which are due to weakened synaptic drive from the auditory nerve during ARHL.

The finding that D-stellate neurons show no change in intrinsic membrane properties among three age groups of mice was unexpected. In contrast, the excitatory bushy neurons in aged mice have depolarized membrane potential, increased membrane input resistance, and decreased threshold current, which lead to enhanced excitability that would counteract the reduced sensory inputs during ARHL [32]. Similarly, aged neurons in the auditory thalamus or the medial geniculate body are also depolarized with enhanced excitability [42]. These observations suggest that different types of central auditory neurons may be differentially affected in their physiological status during ARHL. In particular, excitatory out-projecting neurons might be more susceptible to weakened sensory inputs and homeostatically adjust their intrinsic excitability during aging than local inhibitory interneurons.

Decreased sensory inputs from the auditory periphery during hearing loss is known to be compensated by enhanced gain in the central nervous system [43,44]. Despite unchanged intrinsic membrane properties, aged D-stellate neurons show reduced neural activity to auditory nerve inputs, which are expected to decrease the overall inhibition to their targeted neurons including bushy neurons in the VCN [2,5,8]. Thus, the responsiveness of aged bushy neurons to auditory nerve inputs would be improved by not only the enhanced excitability of themselves [32], but also by the reduced inhibition from the aged D-stellate neurons. Together, physiological changes in both the inhibitory D-stellate neurons and the excitatory bushy neurons should enhance the central gain of the auditory processing in the CN during ARHL. Reduced inhibition from D-stellate neurons during aging is likely accompanied by degraded temporal coding in excitatory CN neurons since normal glycinergic inhibition helps improve the temporal precision of spikes in out-projecting bushy and T-stellate neurons in the VCN [2].

To date, much more focus of ARHL has been on changes in the periphery auditory system [45,46,47,48], especially on the status of hair cells and the connected spiral ganglion neurons [37,49]. In CBA/CaJ mice, loss of outer hair cells as well as spiral ganglion neurons across the entire frequency spectrum of hearing were observed with increased age, which lead to decreased auditory inputs to the central nervous system and elevated hearing threshold during ARHL [37]. The finding that auditory nerve inputs onto D-stellate neurons decrease in synaptic drive with age indicates that auditory nerve central terminals of the surviving spiral ganglion neurons are functionally compromised during ARHL. This is consistent with our previous study that the synaptic transmission at the giant auditory nerve terminal of Endbulb of Held deteriorates with age [33], which leads to decreased firing rate and temporal precision in postsynaptic bushy neurons during ARHL [32]. The findings in this study further suggest that ARHL-associated decline in synaptic function is not just specific to the highly specialized Endbulb of Held synapses, but also occurs at other auditory nerve central terminals in the CN. The fact that the synaptic function observed from the middle age group were intermediate between the young and the aged mice (Figure 6) further indicates that the functional decline of the auditory nerve central synapses is a slow but continuous process during aging, which accompanies the progression of the hearing status of ARHL (Figure 1). 

## 5. Conclusions

This study investigated the physiological changes of the inhibitory D-stellate neurons of the VCN in three age groups of mice. We found that D-stellate neurons maintain stable membrane properties, but decrease in neuronal activity during aging because of weakened synaptic drive from the auditory nerve inputs. We conclude that the reduced activity in aged D-stellate neurons decreases the overall inhibition and enhances the central gain in the CN, which serves as a compensatory mechanism to counteract the reduced sensory inputs from the auditory periphery during ARHL.
